# Efficacy of Intrathecal Morphine in a Model of Surgical Pain in Rats

**DOI:** 10.1371/journal.pone.0163909

**Published:** 2016-10-26

**Authors:** Aurelie Thomas, Amy Miller, Johnny Roughan, Aneesa Malik, Katherine Haylor, Charlotte Sandersen, Paul Flecknell, Matthew Leach

**Affiliations:** 1 Comparative Biology Centre, Institute of Neuroscience, Newcastle University, Newcastle upon Tyne, United Kingdom; 2 School of Agriculture, Food and Rural Development, Newcastle University, Newcastle upon Tyne, United Kingdom; 3 Royal (Dick) School of Veterinary Studies, Edinburgh, United Kingdom; 4 School of Biomedical Sciences, Newcastle University, Newcastle Upon Tyne, United Kingdom; 5 Clinique Vétérinaire Universitaire, Faculté de Médecine Vétérinaire, Université de Liège, Liège, Belgium; Max Delbruck Centrum fur Molekulare Medizin Berlin Buch, GERMANY

## Abstract

Concerns over interactions between analgesics and experimental outcomes are a major reason for withholding opioids from rats undergoing surgical procedures. Only a fraction of morphine injected intravenously reaches receptors responsible for analgesia in the central nervous system. Intrathecal administration of morphine may represent a way to provide rats with analgesia while minimizing the amount of morphine injected. This study aimed to assess whether morphine injected intrathecally via direct lumbar puncture provides sufficient analgesia to rats exposed to acute surgical pain (caudal laparotomy).In an initial blinded, randomised study, pain-free rats received morphine subcutaneously (MSC, 3mg.kg-1, N = 6), intrathecally (MIT, 0.2mg.kg-1, N = 6); NaCl subcutaneously (NSC, N = 6) or intrathecally (NIT, N = 6). Previously validated pain behaviours, activity and Rat Grimace Scale (RGS) scores were recorded at baseline, 1, 2, 4 and 8h post-injection. Morphine-treated rats had similar behaviours to NaCl rats, but their RGS scores were significantly different over time and between treatments. In a second blinded study, rats (N = 28) were randomly allocated to one of the following four treatments (N = 7): MSC, 3mg.kg-1, surgery; MIT, 0.2mg.kg-1, surgery; NIT, surgery; NSC, sham surgery. Composite Pain Behaviours (CPB) and RGS were recorded as previously. CPB in MIT and MSC groups were not significantly different to NSC group. MSC and MIT rats displayed significantly lower RGS scores than NIT rats at 1 and 8h postoperatively. RGS scores for MIT and MSC rats were not significantly different at 1, 2, and 8h postoperatively. Intraclass correlation value amongst operators involved in RGS scoring (N = 9) was 0.913 for total RGS score. Intrathecal morphine was mostly indistinguishable from its subcutaneous counterpart, providing pain relief lasting up to 8 hours in a rat model of surgical pain. Further studies are warranted to clarify the relevance of the rat grimace scale for assessing pain in rats that have received opioid analgesics.

## Introduction

Rodents remain the most commonly used animals for fundamental science and translational medicine [1]. Public perception and acceptance of animal models for biomedical research relies on the respect of fundamental ethical rules, such as the systematic implementation of the 3Rs: replacement, reduction and refinement [2–5]. In particular, refinement, which aims to reduce to a minimum any pain or distress caused by research procedures [6] can be applied to any surgical procedures by providing effective analgesia. The need to adopt this approach is a requirement of the European Directive EU 2010/63/EU (Article 14) [4]. Whilst the vast majority of rodent surgical research procedures are performed under anaesthesia, surveys indicate that the use of perioperative analgesics remains low; e.g. in less than 25% of rats undergoing surgery [7–9]. The main reported reasons for this are concerns over interactions with results of studies or potential negative side-effects from the analgesic themselves, or simply that there was no perceived need for using pain relief as a consequence of an inability to effectively recognise pain [7,8,10].

Morphine, widely used in humans since the early years of the 19^th^ century, is a full agonist of the mu-opioid receptor that is well absorbed under clinically used routes of administration [11–13]. It is commonly used for the prevention and treatment of severe acute and chronic pain in both humans and animals [14,15]. Clinically relevant side effects of morphine in painful rodents are negligible when administered at appropriate doses for a short duration [14]. However, pica has been cited as a reason for withholding morphine and other opioids such as buprenorphine in rats [16,17]. The incidence of pica-type behaviour depends on several factors such as dose, frequency of opioid administration, strain of rat and type of bedding [17,18]. In rodents, respiratory depression, another commonly quoted side effect following opioid use appears to have little clinical significance in rodents [19,20]. Concerns related to the effects of opioids on various physiological systems may be relevant in some specific research areas; for example the immunomodulatory effects of morphine. Morphine use could trigger a series of effects, such as pro-inflammatory variation in the nervous system [21] and altered tumour growth profiles [22,23]. Many of these effects can be minimised by reduction of the dose of morphine administered [23]. For example, following intravenous administration of morphine in humans, only about 0.1% of the total drug administered is present in the central nervous system (CNS) at the time of peak plasma concentration [24]. Reasons for the relative poor penetration of morphine into the CNS include its relatively poor lipid solubility compared to other opioids and rapid conjugation (metabolism) with glucuronic acid. If the same is true in animals, then use of alternative routes of administration could enable effective analgesia to be provided with lower total doses of morphine. Morphine is commonly administered neuraxially (epidural or intrathecal routes) in non-rodent species [25,26]. In humans, the ratio of potencies between intrathecal (IT) and intravenous (IV) routes of administration is 1/200 [27]; i.e. equipotent analgesic effects can be achieved using a dose that is 200 times less if the IT route is chosen.

Techniques for intrathecal injection in rats have been described and although technically more challenging than parenteral routes of injection, could provide a practical means of establishing effective analgesia with a much reduced dose of morphine [28–31]. Intrathecal morphine was shown to alleviate pain in rats using nociceptive assays in the Brennan model of post-operative pain [32], and to restore exploratory behaviour post sub-costal laparotomy [33] and rearing as well as ambulation in a rat model of knee surgery [34]. Intrathecal injection in rats is also widely used to test therapeutic targets [35].

Refinement of research procedures involving animals partly relies on effective ways to prevent and alleviate pain; which requires effective and reliable methods to detect and quantify pain. Considerable progress has been made in developing such techniques in rats, providing assessment methods that are more relevant to the assessment of post-operative pain than basic nociceptive tests. Current widely used approaches measure a range of pain specific behaviours and use these to construct scales for assessing pain (e.g. the Composite Pain Scale) [36]. These scales can be used effectively, but can be influenced by non-specific behavioural effects of opioids such as morphine [37,38]. Facial expressions can also be used to assess pain [39]. Grimace scales have been developed for rodents [40–42], and have been used to refine experimental models [43,44] or assess the efficacy of common analgesic drugs [45,46], However, such scales have been suggested to be influenced by non-specific behavioural effects of opioids [41] and further validation must be carried out to determine if they are a suitable method of pain assessment following morphine administration.

The aims of this study were to assess if intrathecal morphine could provide sufficient analgesia to prevent pain in rats undergoing caudal laparotomy, as assessed by analysis of behavioural changes and the rat grimace scale. We hypothesised that a lower dose of morphine, administered pre-emptively by the intrathecal route would have similar analgesic properties to a routine dose injected SC. We also tested the hypothesis that this lower dose of morphine would have fewer non-specific behavioural effects than morphine administered at higher doses by the SC route.

## Material and Methods

### Ethical statement

All procedures were carried out under Home Office approved project and personal licenses (PPL 60/4431), compliant with the Animals (Scientific Procedures) Act 1986, EU directive (2010/63) and the Animal Welfare, Ethics Review Body at Newcastle University (AWERB).

### Animals and husbandry

Fifty-two female Wistar rats (Charles River Laboratories, Kent, UK) were used in this study (270±14.7g; 61±3 days old). All animals were housed in randomly allocated groups of 3 to 5 rats per cage (RC2 cages, North Kent Plastics Company), provided with environmental enrichment (cardboard tubes, Datesand, Manchester, UK), sawdust substrate (Aspen 4HK, UK) and nesting material (Sizzle nest, LBS technology, Surrey, UK). Food (RM3, SDS Ltd, Witham, UK) and tap water were provided *ad libitum*. Room temperature was 21±2°C, humidity was 55±10%, with a 12h light cycle (7am-7pm). All rats were allowed to acclimatize for 7 days before starting the experiment, during which time the rats were habituated to the laboratory and researchers. The animals were free from any common pathogens in accordance with the FELASA health monitoring recommendations.

### Study Design

This study comprised of two phases. The 1^st^ phase was designed to assess the effect of morphine, delivered subcutaneously (SC) or intrathecally (IT), on behaviour and facial expressions in pain free rats. The second phase was designed to assess similar effects on rats following abdominal surgery. While each phase was performed and analysed separately; the study design and the method of collecting data were identical in each phase. The study design is therefore only described once. Two main operators were involved in this study: the anaesthetist injecting the rats (AT- blinded to the nature of the injection) and the surgeon (PAF, 2^nd^ phase only, blinded to the nature and the site of injection). Three other operators assisted with the recording of behaviour and facial expression data. All 3 operators were blinded to the nature and site of the injection received by the rats (NaCl vs. morphine).

### Treatment groups

Twenty-four and twenty-eight rats were randomly allocated to one of 4 treatment groups in phase 1 and 2 of the study, respectively. All treatment groups are described in Tables 1 and 2. Random allocated was carried out using an online random number generator (www.random.org). Treatment groups 1.1 to 1.4 (1^st^ phase) were control groups designed to assess the possible effect of morphine (SC and IT) on behaviour and facial expression of pain-free rats. Treatment groups 2.1 to 2.4 (2^nd^ phase) were designed to assess the analgesic action of morphine (SC and IT) on acute surgical pain in rats. The morphine used (treatment groups 1.3, 1.4, 2.3, 2.4) was sterile and free of preservative (Morphine sulphate, 1mg.ml^-1^, South Devon Healthcare, Paignton, UK). The NaCl solution used (treatments groups 1.1, 1.2, 2.1, 2.2) was normal (0.9% w/v) and sterile (Braun, Melsungen, Germany). The dose of morphine selected for SC administration is commonly used in rats post-operative analgesia [14,47,48]. The dose for intrathecal injection (0.2 mg.kg^-1^) was selected from previous pilot studies (non-published data) and represents the highest dose and volume (average 54 μg and 54 μl) of morphine that can be administered without causing significant side effects (*i*.*e*. respiratory depression).

**Table 1:** Treatment groups in phase 1 of the study.

**A. 1^st^ Phase**	**Treatment**	**N**	**Drug**	**Route**	**Dose [mg.kg^-1^]**	**Volume [ml.kg^-1^]**
	1.1	6	NaCl 0.9%	SC	N/A	3
	1.2	6	NaCl 0.9%	IT	N/A	0.2
	1.3	6	Morphine	SC	3	3
	1.4	6	Morphine	IT	0.2	0.2

**Table 2:** Treatment groups in phase 2 of the study.

**B. 2nd Phase**	**Treatment**	**N**	**Surgery**	**Drug**	**Route**	**Dose [mg.kg^-1^]**	**Volume [ml.kg^-1^]**
	2.1	7	Sham	NaCl 0.9%	SC	N/A	3
	2.2	7	Laparotomy	NaCl 0.9%	IT	N/A	0.2
	2.3	7	Laparotomy	Morphine	SC	3	3
	2.4	7	Laparotomy	Morphine	IT	0.2	0.2

### Anaesthesia

All treatments were carried under general anaesthesia, between 08.00am and 12.00noon. Anaesthesia was induced in a 7L Plexiglas induction chamber with sevoflurane (inspired fraction of sevoflurane (F_i_Sevo) = 8%, Abbott, Maidenhead, UK) delivered in 4 l.min^-1^ O_2_. After loss of consciousness (assessed by loss of the righting reflex), the rats were transferred from the chamber and anaesthesia maintained using a rodent-size facemask (VetTech Solutions Ltd, Cheshire, UK) with sevoflurane (F_i_Sevo = 2.4% delivered in 1.5l.min^-1^ O_2_). Normothermia was maintained using a homeothermic heat pad (Harvard Apparatus, Kent, UK). Heart rate and hemoglobin saturation in oxygen were monitored using a rodent pulse oxymeter (Physiosuite, Kent Scientific Corporation, Torrington, USA). At the end of the procedure (see below), sevoflurane was discontinued and the rats were allowed to recover for a few minutes on the heat pad (O_2_: 1.5 l.min^-1^) before transfer to an incubator (28±3 deg C) until full recovery of coordinated motor function (10–15 min). The duration of anaesthesia was approximately 5 minutes (no surgery) to 15 min (surgery, see below).

### Injections

**Fig 1:**
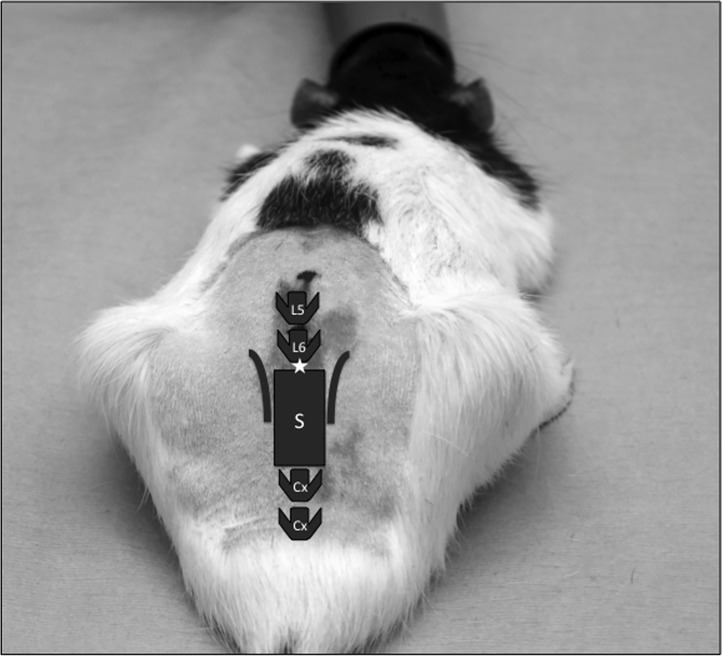
Intrathecal injection in rats: anatomical landmarks and site of injection. Cx: coccygeal vertebrae, L5: fifth lumbar verterbrae, L6: sixth lumbar vertebrae, S: sacrum. ★: injection site (L6-S1).

Regardless of their allocated treatment groups, all rats had the abdomen and lumbosacral area shaved at the start of the maintenance of anaesthesia. The shaved areas were cleaned with a diluted solution of chlorhexidine. Rats allocated to treatments 1.1, 1.2, 2.1, 2.2 were injected subcutaneously in the scruff with a sterile 25G needle and hypodermic syringe. Rats allocated to treatments 1.3, 1.4, 2.3, 2.4 were positioned in sternal recumbency with their pelvic limbs brought under the abdomen as cranially as possible in order to arch the lumbosacral area. The intrathecal injection was performed using aseptic techniques, using a 25G hypodermic needle and an insulin syringe (0.5ml). The injection site was between the last lumbar vertebra and the 1^st^ sacral vertebrae (L6-S1) (Fig 1). The injection was considered successful if one of the 2 following signs were noted: presence of CSF in the needle hub prior to injection and/or twitch of the tail during the injection. If none of these signs were noted, or if blood was visible in the needle hub prior to injection, the needle was withdrawn and another sterile needle was used to repeat the procedure. All intrathecal injections were successful at the 1^st^ or 2^nd^ attempt.

### Surgery

Rats allocated to treatments 2.2, 2.3, 2.4 underwent a caudal laparotomy with bladder wall injection immediately after the injections described above. The procedure is an adaptation from a previously described technique to produce a clinically relevant model of abdominal surgery [36,49]. Briefly, a caudal and midline laparotomy incision (1cm) was performed. The bladder was exposed and its wall injected with 0.1ml of sterile NaCl 0.9% (Braun, Melsungen, Germany) with an insulin needle and syringe (Terumo, Inchinnan, UK). The abdominal muscles were sutured with an interrupted pattern (4.0 Vicryl, Ethicon, Wokingham, UK) and the skin with a subcuticular pattern of the same suture material.

### Behaviour recording

A custom made filming cage (40 x 26 x 28 cm) was used for behavioural recording. The cage did not contain any bedding, food or water. Three vertical sides, floor and ceiling were lined with a matte black film (Fablon) to minimise sensory distraction and reflections. A high definition camera (Sony High Definition HandyCam model HDR-XR155, Sony, Japan) was positioned using a tripod to face the remaining clear Perspex side of the cage. The rats were placed individually in the cage, allowed to acclimatize for 5 minutes and filmed for 10 minutes without an observer being present in the filming area. The box was thoroughly cleaned after filming each animal. Each rat was filmed on the day prior to the procedure (baseline = T_0_) as well as at 1, 2, 4, and 8h post-injection.

#### Phase 1 of the study

An operator blinded to the rats’ treatment scored each 10-minute video clip using open-source software designed to score animal behaviour from video clips (Cowlog) [50]. Behavioural scoring was performed using a validated rat ethogram [36,49,51]. The ethogram is summarised in Table 3. Duration and frequency for each of the defined behaviours were recorded. A Composite Pain Behaviour (CPB) score was calculated by summing the frequency of the pain behaviours for each individual rat at each time-point. Pain behaviours included in the CPB in this phase were: back arching, twitch and stagger/fall.

**Table 3:** Ethogram used for behavioural observations in phase 1. Adapted from references [36,49,51].

	Behaviour	Description
**Pain Behaviours**	Back arching	Vertical stretch as in felines upon waking, including both partial and full arches, while inactive or walking.
	Twitch	Transient involuntary muscular contraction of any body part. Usually occurs while inactive.
	Stagger/ fall	Rapid transition to crouch from high or low rear. Also, falling during grooming while crouched.
	Belly Pressing	Rubbing the laparotomy site purposefully across the floor of the cage
	Writhe	One or more contractions of the abdominal muscle on either side of the stationary or moving animal, lasting until the abdomen relaxes.
**Ambulatory Behaviours**	High rear	Bipedal stance, fully erect posture
	Down to partial	Downward movement from high rear to partial rear
	Partial rear	Bipedal stance, low or half–erect posture
	Down to floor	Downward movement from either high rear, partial rear, jump or climb to quadrupedal contact with the cage floor
	Walk	Quadrupedal ambulatory movement
	Inactive	Still, no on–going activity
	Jump	Transient vertical projection of entire body, usually follows high rear
	Climb	Hind paws not in contact with the cage floor, usually follows high rear or precedes jump
	Other	Any behaviour not specified. See below.
**Grooming Behaviours**	Groom head	Grooming, licking or scratching head
	Groom limbs	Grooming, licking or scratching limbs
	Groom abdomen	Grooming, licking or scratching abdomen, includes paying attention to wound site or abdominal shaved area
	Groom back	Grooming, licking or scratching back, includes paying attention to lumbar shaved area
	Groom tail	Grooming, licking or scratching tail
**Other Behaviours**	Investigate	Vigorously sniff, explore, or inspect something. Recorded only when done in isolation (animal usually inactive or between ambulatory behaviours). Usually investigate a cage item but can sniff the air.
	Chew	Bite or gnaw part of the cage. Usually cage walls but can include masticating a cage item such as a piece of substrate
	Eat	Ingestion of an item
	Stretch	Horizontal elongation of body. Usually precedes and follows walking.
	Shake	Instantaneous shuddering lateral whole body movements
	Lift hind leg	Transient upward movement of one hind paw or hind leg so that it is no longer in contact with the floor
	Squint	Partial closure of eyes
	Teeth chatter	Vertical or lateral mouth/ jaw movements or teeth grinding in the absence of any obvious item to be chewed or eaten
	Dig	Digging on cage floor, usually with forepaws. Usually follows investigate
**Miscellaneous ‘other’ behaviours**		On each occasion ‘other’ was scored, a note was made of this behaviour that was being performed by the rat.

#### Phase 2 of the study

One treatment-blinded operator (KH) (blinded to time point and treatment) was responsible for all behavioural assessments. Briefly, the video was played in real time, and the operator scored the incidence of certain specific behaviours. Pain behaviours included in the CPB score in this phase were: back arching, twitch, stagger/fall, and belly pressing. Behaviour scoring of 5 minute per rat, per time point was carried out.

### Rat Grimace Scale

Following video recording for behavioural analysis, rats were placed in a photography box for a period of 5 minutes and photographed using a high definition camera (Casio EX-ZR100, Casio Computer Co., Ltd., Japan) by a treatment-blinded observer. The photography box consisted of two matte black walls and two clear Perspex walls (27cm x 19cm x 17cm). Rats were photographed on every occasion they directly faced the camera, apart from when grooming in accordance with the method described by Sotocinal and colleagues [40]. The rats were then returned to their home cages. A treatment-blinded observer recorded any unexpected event or complication related to picture acquisition. The box was thoroughly cleaned and dried after photogrpahing each animal. All out-of-focus or out-of-frame images were manually deleted. The remaining images were cropped, leaving only the face of the rat in view to prevent bias due to body posture [41]. Using a random number generator, one image per rat, per time point was selected. Using the random number generator again, the selected images were re-ordered and inserted into a custom designed excel file for scoring. Nine trained observers who were blinded to the experimental details, design and purpose of the study scored each photograph for the four facial action units (FAUs) comprising the RGS as described by Sotocinal and colleagues [40]. For each image, the 4 individual FAUS: orbit tightening, nose / cheek flattening, ear changes and whisker change were scored using a 3-point scale (0 = not present, 1 = moderately present, 2 = obviously present). The nine scorers consisted of 5 veterinarians (including 2 with experience of working with rodents) and 4 scientists with no experience of working with rodents.

All rats were euthanized using a rising concentration of inhaled C0_2_ (filling rate: 20% of the chamber volume per minute) following recording of the last set of data, 8h post-injection, in accordance with current guidelines and legislation [4,52].

### Statistical Analysis

All statistical analyses were conducted using SPSS (SPSS Inc. Chicago, USA). A sphericity test was performed to verify the parametric nature of the data after which repeated measures ANOVA with ‘time’ as a within subjects factor and ‘treatment’ as a between subjects factor were performed. Pairwise comparisons were examined with post hoc analysis (Bonferroni). Data from the 1^st^ and 2^nd^ phases of the study were analysed separately. Differences were considered significant if p<0.05.

The RGS (0–8) score was obtained by averaging the scores (0–2) obtained for each of the 4 action units (AU) for each analysed image. Time effects within treatments were assessed with a Friedman’s test. Treatment effect at each time points were identified using a Kruskall-Wallis test and further investigated with Mann-Whitney U Tests and Bonferroni corrections for multiple comparisons. Comparisons of equivalent treatments across study phases (*i*.*e*. treatments 1.1 vs. 2.1; 1.2 vs. 2.2; 1.3 vs. 2.3; 1.4 vs. 2.4) were also performed using Mann-Whitney U Tests with adjusted Bonferroni corrections for multiple comparisons. Finally, the level of consistency (reliability) between scorers was illustrated with Intraclass Correlation Coefficients (ICC) for each action unit as well as for the total RGS score. In all cases, differences were considered significant if p<0.05.

### Power of the study

Retrospective assessment of the power of the study (2^nd^ phase only) was performed using G*Power (Softnews NET SRL, Romania).

For the behavioural analysis (CPB), the power exceeded 0.8 with respect to differences between time points and with respect to differences between the saline surgical group and both morphine groups. The power with which to detect differences between the morphine groups was approximately 0.4. Similar results were obtained for the RGS (within subjects, power >0.8; within morphine groups: 0.33).

## Results

### Phase 1: treatments 1.1 to 1.4

#### Effect of morphine (SC and IT) on the behaviour of pain-free rats

There was no significant treatment effect on the behaviour of rats. Neither morphine administered SC or IT, or NaCl significantly affected the frequency and duration of behaviours identified in the rat ethogram

**Fig 2:**
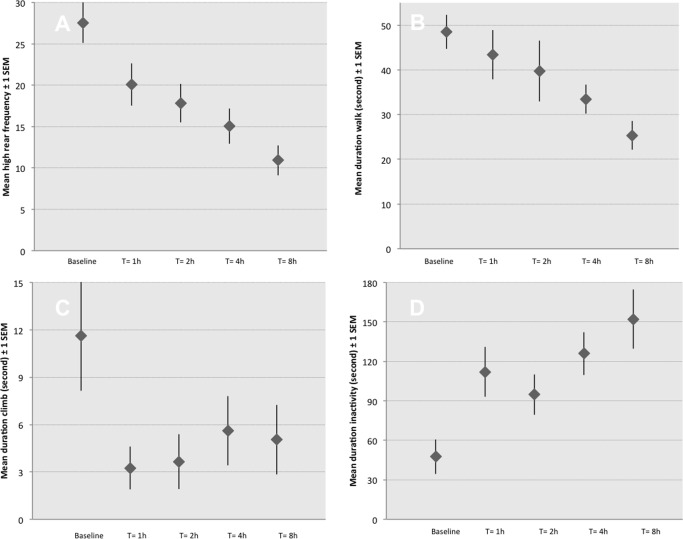
Representation of mean duration or frequency of the most commonly observed behaviours in pain-free rats (1^st^ phase study) ± 2 standard error to the mean. N = 24, pooled data for treatment groups 1.1 to 1.4. There was no significant treatment effect between groups. **A**: significant decrease of rearing frequency over time (p<0.001); **B**: significant decrease of time spent walking over time (p<0.001); **C**: Time spent climbing is significantly higher at baseline than at any further time point (P = 0.036); **D:** significant increase of the time spent inactive (p = 0.001).

Time had a significant effect on some of the behaviours across all treatments (1.1 to 1.4), as illustrated in Fig 2. As such, the frequency of high rears significantly decreases (p<0.001), the duration of walking decreased (p<0.001), the duration of climbing behaviours were significantly higher at baseline compared to other time points (p = 0.03). Lastly, the duration of inactivity increased over time with duration of inactivity being significantly lower at baseline compared to other time points (p<0.001). There was no significant effect of time on any of the other analysed behaviours. No pain specific behaviours were detected.

#### Effect of morphine (SC and IT) on Rat Grimace Scale (RGS) score in pain-free rats

**Fig 3:**
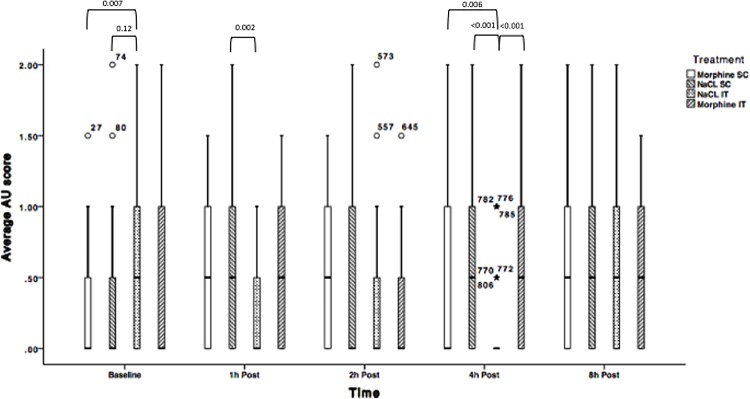
Average Action Unit RGS scores for treatments 1.1 (NaCl SC); 1.2 (NaCl IT); 1.3 (Morphine SC) and 1.4 (morphine IT) over time. N = 24 (Solid line = median, box = 1^st^ and 3^rd^ quartiles, whiskers = minimum and maximum, ★,○ = Outliers). AU: Action Unit. *P*-values are indicated where differences are significant.

Average AU RGS results are documented in Fig 3 and Table 4.

**Table 4:** Within-subjects comparison between post treatment time-points with baseline with associated *p*-values for differences where significance was found (phase 1 of the study). Differences are significant if p<0.05. AU: Action Unit; SC: Subcutaneous; IT: Intrathecal; ND: No significant difference.

**Average AU Scores- NaCl SC**				
Time point	1h	2h	4h	8h
Baseline	<0.001	0.002	0.003	0.001
**Average AU Scores–NaCl IT**				
Time point	1h	2h	4h	8h
Baseline	ND	ND	0.001	ND
**Average AU Scores- Morphine SC**				
Time point	1h	2h	4h	8h
Baseline	0.007	0.007	ND	0.007

Average AU RGS scores were significantly different across treatments at baseline, 1 and 4h post injection. At 1h post-injection, rats receiving intrathecal NaCl had a significantly lower RGS score than rats having received NaCl subcutaneously (p = 0.002). At 4h post-injection, total RGS was significantly lower in rats allocated to the NaCl IT group compared to: NaCl SC (p<0.001) and the morphine groups (morphine SC: p = 0.006; morphine IT: p<0.001) compared to their respective control groups.

Average AU RGS score also significantly increased over time in pain-free rats for all treatments except morphine intrathecally (Fig 3 and Table 4).

### Phase 2: treatments 2.1 to 2.4

#### Analgesic properties of intrathecal morphine in rats subjected to acute surgical pain

**Fig 4:**
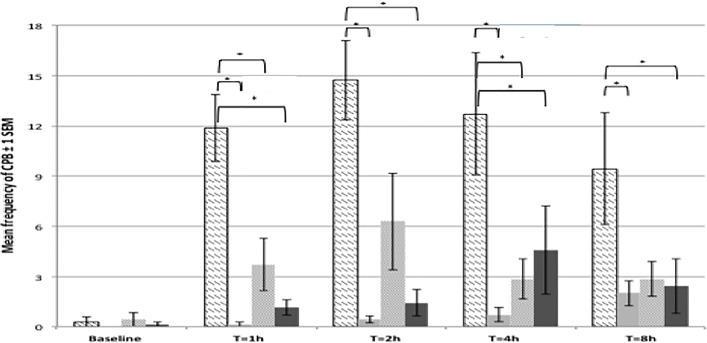
Mean frequencies for Composite Pain Behaviour (CPB) scores across treatments and time points. Data presented ± 2 standard error of the mean. N = 28. Significant differences (*) are explained in the text and Table 5.

The surgical model used in the present study (caudal laparotomy with bladder wall injection) resulted in the presence of previously validated, specific pain behaviours such as writhing, fall/stagger, twitches, and belly pressing. The incidence of back arching was very low and was therefore removed from the analysis. The composite pain behaviour (CPB) score was therefore obtained using the mean frequencies of the following behaviours: writhing, fall/stagger, belly pressing and twitches (Fig 4). CPB score was very low and not significantly different between treatment groups at baseline. Rats allocated to undergo surgery and receive an intrathecal injection of NaCl showed had a significantly higher CPB score than other rats allocated to the sham group at all postoperative time points (Table 5). The peak of CPB was observed at 2h post surgery.

**Table 5:** Composite Pain Behaviour scores and associated *p*-values for phase 2 of the study.

Time	Baseline				1h post surgery				2h post surgery				4h post surgery				8h post surgery			
Treatment	NaCl IT, Sx	NaCl SC, Sh	Mor IT, Sx	Mor SC, Sx	NaCl IT, Sx	NaCl SC, Sh	Mor IT, Sx	Mor SC, Sx	NaCl IT, Sx	NaCl SC, Sh	Mor IT, Sx	Mor SC, Sx	NaCl IT, Sx	NaCl SC, Sh	Mor IT, Sx	Mor SC, Sx	NaCl IT, Sx	NaCl SC, Sh	Mor IT, Sx	Mor SC, Sx
**NaCl IT, Sx**	/	ND	ND	ND	/	<0.001	0.006	<0.001	/	<0.001	ND	0.039	/	0.001	0.011	0.045	/	0.013	ND	0.035
**NaCl SC, Sh**	ND	/	ND	ND	<0.001	/	ND	ND	<0.001	/	ND	ND	0.001	/	ND	ND	0.013	/	ND	ND
**Mor IT, Sx**	ND	ND	/	ND	0.006	ND	/	ND	ND	ND	/	ND	0.011	ND	/	ND	ND	ND	/	ND
**Mor SC, Sx**	ND	ND	ND	/	<0.001	ND	ND	/	0.039	ND	ND	/	0.045	ND	ND	/	0.035	ND	ND	/

P values below 0.05 indicated a significant difference. ND: No significant difference (p>0.05). IT: Intrathecal, Mor: Morphine, NaCl: Saline, SC: Subcutaneous, Sh: Sham, Sx: Surgery. See Table 2 for full information on treatments

Morphine, administered subcutaneously or intrathecally, resulted in significantly lower CPB scores compared to control rats (NaCl IT, surgery) at all time points. CPB scores in morphine-treated rats (either SC or IT) were not significantly different to CPB scores in the sham surgery group. There was no significant difference in the CPB score of rats undergoing surgery and receiving morphine either subcutaneously or intrathecally, at any time point. See Table 5 for the full list of significant differences between treatments at all time points.

**Fig 5:**
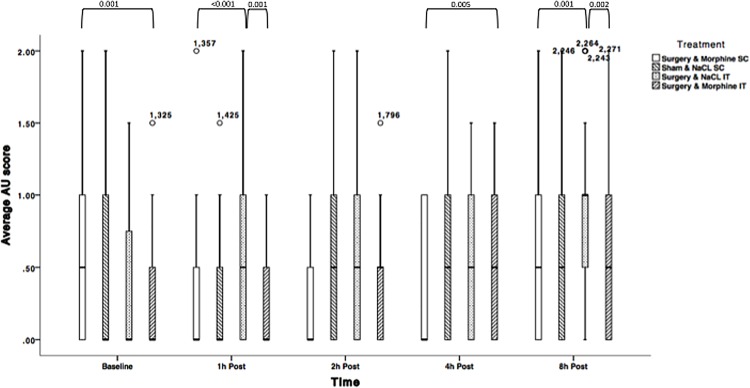
Average Action Unit RGS scores for treatments 2.1 (NaCl SC + sham surgery); 2.2 (NaCl IT + surgery); 2.3 (morphine SC + surgery) and 2.4 (morphine IT + surgery) over time (Solid line = median, box = 1^st^ and 3^rd^ quartiles, whiskers = minimum and maximum, ★,○ = Outliers). N = 28. *P*-values are indicated where differences are significant (<0.05).

Average AU RGS scores for rats undergoing surgery and receiving NaCl IT significantly changed over time: average AU scores were significantly lower at baseline compared to 8h post laparotomy (p<0.01), but not at other time points. Similar results were obtained for rats undergoing sham surgery. Average AU scores for rats undergoing surgery and receiving morphine remained similar over time if the drug was administered subcutaneously, but were higher 8h post surgery if morphine was injected IT (p<0.001). Detailed results are displayed in Fig 5 and Table 6.

**Table 6:** Within-subjects comparison between post treatment time-points with baseline with associated *p*-values for differences where significance was found (Phase 2 of the study). Differences are significant if p<0.05. AU: Action Unit; SC: Subcutaneous; IT: Intrathecal; ND: No significant difference.

**Average AU Scores- NaCl SC, Sham**				
Time Point	1h	2h	4h	8h
Baseline	ND	ND	ND	0.010
**Average AU Scores–NaCl IT, Surgery**				
Time Point	1h	2h	4h	8h
Baseline	ND	ND	ND	<0.001
**Average AU Scores- Morphine IT, Surgery**				
Time Point	1h	2h	4h	8h
Baseline	ND	ND	<0.001	<0.001

Morphine-treated rats (SC and IT) undergoing surgery, displayed significantly lower total RGS scores than control rats (NaCl IT) 1h post-surgery (p<0.001, p = 0.001 respectively). Similar pattern of significant differences were identified 8h post-surgery (p = 0.001–0.002). RGS scores were not significantly different between the morphine groups at 1, 2, and 8h postoperatively. RGS scores were not significantly different across treatments 2h post laparotomy. At 4h post laparotomy; RGS scores were not significantly different between rats allocated to the sham surgery groups and those allocated to the surgical groups and intrathecal NaCl.

Average AU RGS scores across treatments were significantly different at baseline between rats allocated to receive morphine SC and (p = 0.01). No other significant differences were noted between groups at baseline.

### Reliability of RGS scoring

The reliability of both the individual AUs and the overall RGS scoring across all 9 scorers was high with intraclass correlation (ICC) values for the individual AUs ranging from 0.69 (nose/cheek flattening) to 0.94 (orbital tightening) and a value of 0.91 for the overall RGS score (P<0.0.001 for all comparisons) (Table 7).

**Table 7:** Inter-rater reliability for the Rat Grimace Scale (2^nd^ phase of the study).

	ICC (average)	95% CI	P
**Overall RGS**	0.913	0.893–0.931	P <0.001
**Orbital tightening**	0.940	0.929–0.951	P <0.001
**Ear positioning**	0.880	0.857–0.901	P <0.001
**Nose and cheek flattening**	0.688	0.626–0.743	P <0.001
**Whisker change**	0.796	0.750–0.837	P <0.001

Intraclass correlation coefficient (ICC) calculated for multiple (average) raters (n = 9).

## Discussion

This novel study demonstrates the effects of morphine injected intrathecally via direct lumbar puncture in pain-free rats and in a model of acute surgical pain. In addition, this study represents the first use of the Rat Grimace Scale to assess the analgesic properties of IT morphine.

### Effects of morphine SC and IT on the behaviour and facial expression of pain-free rats

The 1^st^ phase of the study was designed to determine whether morphine, administered SC or IT, influenced behaviour or rat grimace score (RGS) of pain-free rats. At the doses chosen in this study morphine, injected subcutaneously or intrathecally, did not affect the frequency and duration of behaviours used in a validated rat ethogram [36]. All changes in behavioural scope and pattern in the second phase of the study, in particular behaviours included in the CPB score, can therefore be attributed to the acute surgical pain caused by caudal laparotomy and bladder injection. This finding is contrary to the common belief that opioids alter the behaviour of pain-free rodents [38]. Morphine as well as other opioids do influence the behaviours of pain-free rats, but this has generally been reported when the doses used are much greater than those recommended for clinical use in rats [14,38], or when opioids are administered repeatedly for example in models of opioid tolerance [53,54]. The activity of all rats decreased over time, regardless of the drug received (NaCl or morphine). This reflects either insufficient habituation to the recording box (5 min) prior to the recording (novelty effect) during the early time points; or habituation of the rats, and possible boredom after several recordings in quick succession (a total of 5, 10 min long, behaviour recording sessions) [55,56].

The effect of morphine and the route of administration on pain-free rats were not easily interpreted using RGS. Average AU scores increased over time for all pain-free treatment groups, with the exception of intrathecal morphine. Each of the behavioural recording session was followed by another 10 min long session in a smaller Plexiglas box for RGS scoring. Whilst activity was not recorded in the RGS box, it was observed that the rats were also more inactive over time during the RGS sessions. Several rats were seen to remain immobile for a large part of the RGS session, and appeared disinterested by their surroundings. Such observations seem to correlate with the overall activity patterns (*i*.*e*. inactivity and walk). In the authors’ experience, it would be reasonable to assume that inactive rats placed in a familiar environment could intermittently show signs of drowsiness. Given that orbital tightening is a natural consequence of drowsiness, then an increase in RGS score over time within all treatment groups could have been expected. One might argue that the sedative properties of morphine [14,57,58] could have further accentuated this expected effect, as sedation has already been shown to increase grimace scale scores [59]. While pain-free rats having received NaCl SC had a significantly lower RGS at baseline than at other time points; this pattern was not repeated in the NaCl IT group.

Interestingly, RGS scores for pain-free rats receiving morphine IT did not change significantly over time, despite the same decrease in activity shown by control rats. Intrathecal morphine may therefore have an effect on facial expression of pain-free rats. Typically, pain-free rats receiving IT morphine displayed seemingly wider and slightly more protuberant eyes, than in control rats. This facial appearance of pain-free rats receiving morphine was documented as exophthalmos by some of the RGS treatment-blinded scorers. Morphine is known to cause mydriasis and exophthalmos in pain-free rats [60–62], but this effect was not analysed for significance in our study. Exophthalmos, which is not taken into account by the RGS, might have counteracted any possible orbital tightening from somnolence in the later time points.

Lastly, pain-free rats receiving morphine SC had a significantly higher RGS score 1, 2 and 8h post injection. This could be explained by the sedative properties of morphine [14,57,58] causing some additional degree of orbital tightening. However, if any sedation occurred, this was not reflected by behaviour changes 1h post-injection.

### Analgesic properties of intrathecal morphine for acute surgical pain

The pain caused by the model chosen for this study (caudal laparotomy with bladder wall injection) has been well documented using composite pain behaviour scoring [36,63]. This surgical procedure remains widely used in orthotopic models of bladder cancers in our institution and elsewhere [64,65]. The presence of detectable pain was confirmed by the significant increase in composite pain behaviours in the positive control group up to the last recorded time point, 8h post surgery. This is in line with previously reported pain mediated behavioural alteration, where laparotomy was associated with an increase of pain specific pain behaviour for up to 6.5h [51]; as well as non-specific behavioural alteration for up to 24h [66].

Morphine administered intrathecally via direct lumbosacral puncture significantly attenuated composite pain behaviours (1 and 4h postoperatively) and rat grimace scores (1 and 8h post surgery), and therefore seems to alleviate acute postsurgical pain caused by caudal laparotomy. Morphine IT was mostly indistinguishable from subcutaneous administration based on RGS scoring, but analysis of the composite pain behavior score suggested that SC morphine might provide uninterrupted analgesia for up to 8h.

The duration of action of morphine administered subcutaneously (8h) was longer than expected since morphine SC is usually considered to provide effective post-operative analgesia for no more than 2-4h [14,67,68], with a peak of analgesic and anti-hyperalgesic activity 45–60 min post administration [67]. Morphine administered intrathecally is expected to provide long lasting analgesia in people [69] and animals [70–72]. While neuraxial morphine was shown to have a duration of action of 21h in primates ***[70]***, and up to 24h in dogs and cats [71,72], intrathecal morphine has been associated with markedly shorter duration of action in rats. Most studies undertaken in rats reported a duration of action of approximately 120 min [32,33,73,74]. Results obtained in the present study suggest that intrathecal morphine in rats, might have a longer duration of action. Three key elements of our study design could explain this difference from previous studies. Firstly, the present study documents analgesic properties of morphine in the context of acute surgical pain, using specific pain behaviours validated for post-laparotomy pain in rats. Most other studies assessed the anti-nociceptive properties of morphine using various nociceptive tests (e.g. Von Frey, Hargreaves). Secondly, in our study, in order to assess the practicality of intrathecal administration of post-operative pain management, morphine was administered without prior surgical implantation of a spinal catheter. A spinal catheter is widely used in pharmacological or toxicological studies to facilitate multiple injections of a substance. The presence of the catheter may be associated with chronic inflammatory pain, alteration of rats’ behaviours, and intrinsic variation in the pharmacological properties of some molecules, including morphine [33,75]. Thirdly, the dose used in the present study (0.2 mg.kg^-1^, IT, i.e. average 54μg per rat) was higher than doses used in most anti-nociceptive studies (e.g. 0.16 to 10 μg per rat) [28,29,32,33]. When selected morphine doses were higher, anti-hyperalgesic properties of intrathecal morphine was anecdotally documented to last for up to 4h [76].

The dose chosen was based on pilot studies conducted in our laboratory (unpublished data) and represented the highest dose that could be administered intrathecally without producing clinical and behavioural side effects. We ensured that the total amount remained below 150μg per rat, the intrathecal dose reported to trigger hyperalgesia in rats [77]. A total dose of 0.2 mg.kg^-1^ in rats is also higher than intrathecal doses commonly used in human analgesia [25,27,78,79], even after applying allometric scaling [80]. Further studies would be required to identify the lowest intrathecal dose required to inhibit composite pain behaviours in rats undergoing laparotomies and other surgical procedures involving the abdomen and the pelvic limbs. Lastly, the effects of a low morphine dose (54μg or below) administered intrathecally on the immune system and cancer models is unknown to date, but would be expected to be lower than systemic doses previously investigated [22,23].

### Relevance of the rat grimace scale for assessing pain in opioid medicated rats

A range of different approaches have been developed to assess the degree of pain experienced by rats undergoing surgical and other traumatic pain, with the view of refining the procedures and/or improving the relevance of translational studies. Beyond nonspecific and retrospective methods (such as weight loss, biochemical stress markers etc.), two prospective pain assessment methods are currently most relied upon. Behaviour-based assessments of pain have been developed for both rats and mice following surgery and other traumatic procedures, and use either the appearance of abnormal behaviours, or the change in the frequency of normal behaviour patterns to score pain [11,49,51,81]. There remain a number of limitations to using behaviour to assess pain in animals, such as possible confounding factor of opioid analgesic on the behaviour of pain-free animals [51] and the specific behavioural responses to painful stimuli varying markedly depending on the nature of the surgical procedure (abdominal-based or other) [11,49,51,81]. The use of facial expressions to assess pain [40] was suggested to overcome some of the above difficulties. Variation of facial expression during painful events was codified as the Rat Grimace Scale (RGS), validated for the assessment of acute surgical and nociceptive pain [40,43,82], and used to assess the efficacy of commonly used analgesics in rats [45]. Amongst the proposed advantages supporting the use of RGS was the lack of a confounding effect of opioids on the facial expression of pain-free animals. The present study suggests that opioids may have an effect on facial expression of pain-free rats (overall decrease of RGS scores over time) caused by a degree of opioid-mediated exophthalmos, but no behavioural effect over time. These findings would benefit from further investigation.

In rats subjected to acute surgical pain, unexpected findings have weakened our interpretation from RGS results. RGS scores in pain free and opioid free rats were expected to be lower than were observed in this study [40] and similar across treatment groups. Importantly, significant differences were detected amongst treatment groups at baseline during both phases of the study. This major inconsistency existed despite overall excellent inter-scorer reliability of the scoring method (ICC 0.913). Three factors either in isolation or combination may have contributed to such findings. Firstly, in mice, baseline mouse grimace scale scores were shown to differ depending on the strain, sex, and methods used for the scoring of facial action units (retrospective vs. prospective scoring) [83]. While variation of baseline scores across strains in Wistar rats, or the impact of retrospective RGS scoring on still images are yet to be documented in a specific study, significant differences amongst baseline RGS scores between treatment groups were unexpected. Rats (strain, age, sex, age), operators, time of the days and methodology used was similar between both study phases. However, this requires further study as the above finding is based on a single study in 6 mice. A second reason that potentially contributed to the differences in RGS scores at baseline between rats could, more simply, be a cohort effect. All rats were bred by the same supplier, were of a similar age, strain, and received equivalent husbandry in our laboratory. In addition, group housing and random allocation of each rat to his treatment group would be expected to minimize the likelihood of such cohort effect. Nonetheless, the statistical analysis used for this study compensated for any possible cohort effect since using a within-subjects design accounts for the variation between individuals when comparing across time points. Thirdly, a relatively short yet consistent habitation period was used in these studies (5 min), and therefore it is possible that the novel nature of the box used contributed to the variability of facial expressions of the rats, possibly causing inadvertent false positive scoring at baseline that were reduced with repeated exposures. This effect has however not been noted in other studies using grimace scales when similar habitation times [59] or different [82] habituation times were used.

While the overall power of our studies was acceptable (>0.8), the lower power observed between the morphine comparisons should be taken into account when interpreting the results of this pilot study. These comparisons bear further investigation in a future study. This pilot study will provide more accurate information for the sample size determination of future studies than was available when this pilot study was undertaken.

Only female rats were used in this study. Pain perception and analgesic properties of opioid are influenced by gender in rats. Briefly, female rodents are more sensitive than males to noxious stimuli and have lower levels of stress-induced analgesia, whereas male rodents generally have stronger analgesic responses to mu-opioid receptor agonists than females [84,85]. In spite of this, gender was not found to significantly influence specific pain behaviours in similar surgical models of pain in rats [49], and no sex-differences were reported in the original RGS paper [40]. We chose to use single sex female groups and minimise the number of rats involved in this study. Further studies would be required to characterise gender-based difference in the analgesic properties of intrathecal morphine.

## Conclusion

Findings from the present study suggest that administration of intrathecal morphine by percutaneous injection may represent an effective way of providing long lasting pain relief in rodents subjected to caudal laparotomy and bladder wall injection. Intrathecal morphine (0.2 mg.kg^-1^) may provide comparable analgesia to the subcutaneous route (3 mg.kg^-1^) using less that a 10^th^ of the dose required for subcutaneous administration. As a result, the intrathecal route of administration may reduce concerns related to the non-specific effects of opioids when these agents are used to alleviate pain in rodents used in a range of different areas of biomedical research. Further studies would be required to better characterize the effects of such reduced morphine dose on individual experimental outcomes.

While the RGS may have advantages for the assessment of pain in rats compared to scoring of composite pain behaviours, morphine may impact facial expression of pain-free rats and influence use of the RGS in opioid medicated rats. The variation in detection of analgesic effects between the RGS and the CPB score support the use of both techniques, as complementary measures of the behavioural changes induced both by analgesics and post-surgical pain.

## Supporting Information

S1 File Rat Grimace Scale scores and behavioural dataset. (XLSX)
